# Targeting and Monitoring Acute Myeloid Leukaemia with Nucleophosmin-1 (*NPM1*) Mutation

**DOI:** 10.3390/ijms24043161

**Published:** 2023-02-05

**Authors:** Lynn Chin, Chantelle Ye Gwen Wong, Harinder Gill

**Affiliations:** Department of Medicine, School of Clinical Medicine, The University of Hong Kong, Hong Kong, China

**Keywords:** acute myeloid leukaemia, nucleophosmin-1, targeted therapy

## Abstract

Mutations in *NPM1,* also known as nucleophosmin-1, B23, NO38, or numatrin, are seen in approximately one-third of patients with acute myeloid leukaemia (AML). A plethora of treatment strategies have been studied to determine the best possible approach to curing *NPM1*-mutated AML. Here, we introduce the structure and function of NPM1 and describe the application of minimal residual disease (MRD) monitoring using molecular methods by means of quantitative polymerase chain reaction (qPCR), droplet digital PCR (ddPCR), next-generation sequencing (NGS), and cytometry by time of flight (CyTOF) to target *NPM1*-mutated AML. Current drugs, now regarded as the standard of care for AML, as well as potential drugs still under development, will also be explored. This review will focus on the role of targeting aberrant *NPM1* pathways such as BCL-2 and SYK; as well as epigenetic regulators (RNA polymerase), DNA intercalators (topoisomerase II), menin inhibitors, and hypomethylating agents. Aside from medication, the effects of stress on AML presentation have been reported, and some possible mechanisms outlined. Moreover, targeted strategies will be briefly discussed, not only for the prevention of abnormal trafficking and localisation of cytoplasmic NPM1 but also for the elimination of mutant NPM1 proteins. Lastly, the advancement of immunotherapy such as targeting CD33, CD123, and PD-1 will be mentioned.

## 1. Introduction

Due to the large proportion of AML patients exhibiting *NPM1*-mutations, targeting *NPM1* is a constant and ongoing area of research, and the wide variety of drugs available are dependent on the types of co-mutations present in *NPM1*-mutants. Despite various clinical trials and research of new agents targeting *NPM1*-mutants, about 50% of patients either relapse or unfortunately die due to the progression of the disease, and this is often the case in patients who are unable to withstand the clinically aggressive nature of AML, nor the harshness of intensive chemotherapy required for disease remission. Seeing as various cellular pathways are affected by *NPM1* mutations, various targeted approaches are being investigated to tackle the challenges in treating relapsed and refractory AML patients. Some examples of *NPM1*-targeting approaches include DNA modulation, ribosome biogenesis, protein deregulation, mitochondrial-mediated cell death, and antibody-mediated treatments.

With the current understanding of *NPM1*-mutant detection, haematologists and medical governing bodies are implementing the use of MRD, also known as a measurable residual disease, for AML patients. The use of MRD can act as a prognostic tool for patients’ treatment, especially during the post-transplantation stage, allowing for a better understanding of the disease progression and prediction of survival outcomes.

Within this review, an overview of the structural changes influencing the functionality of cells is given, followed by *NPM1*-mutated AML risk stratification, monitoring of NPM1 mutants by MRD, and finally targeting NPM1-mutations for treatment of AML patients.

## 2. Functional Role of *NPM1*

### 2.1. Genetic Characteristics

**Figure 1:**
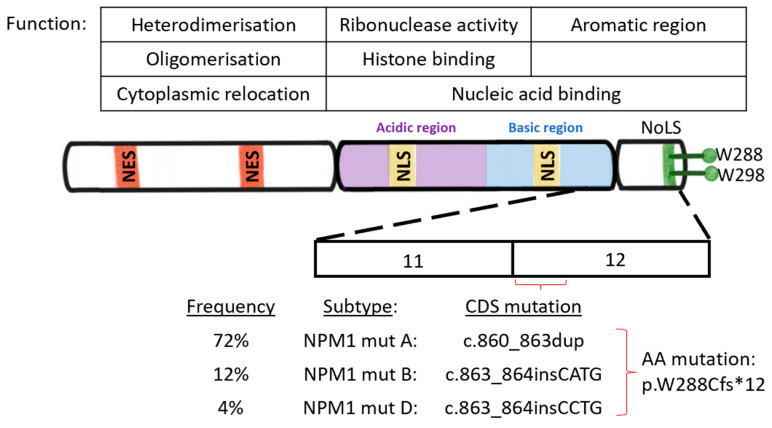
Genetic and functional characteristics of wild-type NPM1 protein. NES: Nuclear export signals, NLS: nuclear localisation signal, NoLS: Nucleolar localisation signal. Type A, B and D NPM1 mutations (denoted as NPM1-mut A, B, and D) with corresponding frequencies found in patients with *NPM1*-mutated AML: CDS mutation (the change that has occurred in the nucleotide sequence) leads to a frameshift, therefore, abolishing tryptophan (W) W288 and W298 at the c-terminal domain (usually present in wild-type NPM1 protein). AA mutation: amino acid mutation of W288Cfs*12 (tryptophan 288 to cysteine frameshift at exon 12).

*NPM1* is localised to chromosome 5q35 and consists of 12 exons. Specifically, NPM1 contains the N-terminal core domain, acidic domain, and nuclear localisation signal domain are found to be highly conserved among each protein of the NPM family, indicating that all three proteins have a similar functional role. However, the less conserved C-terminus, on the other hand, suggests an alternative role for NPM1. It is highly conserved between metazoans and when mutated can result in leukaemic-initiating activity [1]. The *NPM1*-mutated gene is a dominant negative, haploid insufficient mutation that is generally limited to exon 12 [2,3], although rare occurrences have been reported in other regions such as exon 5, 9, and 11 [4,5]. *NPM1*-mutated AML is mostly categorized into type A, B, or D according to a 4 base-pair insertion sequence at the 288th amino acid position (rs587776806) [6]. Type A (sequence: TCTG) is the most frequent, comprising of approximately 72% of *NPM1*-mutated AML cases. Types B (sequence: CATG) representing ~12% and D (sequence: CCTG) with ~4% are less frequent but more so than all other types which represent less than 1% of the *NPM1* mutated population (Seen in Figure 1) [6,7]. The frameshift resulting from *NPM1* mutant insertions causes a change in the C-terminal domain (CTD) amino acid sequence from DLWQWRKSL to DLC(L/M)AVEEVSLRK, consequently replacing one or both tryptophan residues that are required for nucleolar localisation signaling with a nuclear export signal (NES) motif, i.e., LXXX(V/L/F/C/M)XXVXL, where X stands for any residue [2,3,6,7]. Since NPM1 proteins interact with a shuttling receptor XPO1, also known as Exportin, the mutant protein is exported at rates exceeding that at which it is imported, ultimately leading to the delocalisation of mutant NPM1 to the cytoplasm [3]. Wild type (WT) NPM1 is also shuttled out of the nucleus in the process, as NPM1-mutant and NPM1-WT form heterodimers to be co-exported [8].

**Figure 2:**
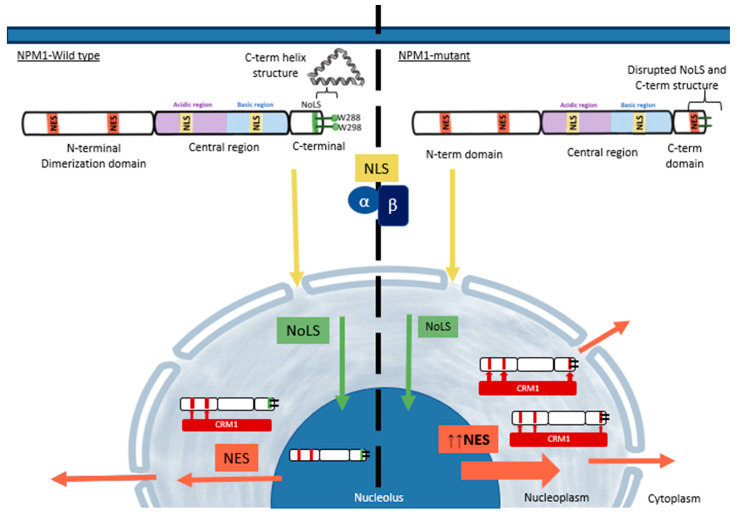
NPM1 wild-type protein, and NPM1-mutant protein structural influences in nucleo-cytoplasmic relocation. Acidic regions influence binding to histone proteins, NLS: nuclear localisation signal binds to importin alpha and beta causing nuclear localisation, NoLS: Nucleolar localisation signals transport NPM1 to the nucleolus, W288, W298: Tryptophan stabilises NoLS and form a C-term helix structure, NES: Nuclear export signals interact with XPO1/CRM1 (chromosomal maintenance 1) protein resulting in an increase in cytoplasmic NPM1.

Modulation of the NPM1 structure is governed mainly by post-translational modifications including phosphorylation, SUMOylation, and ubiquitination. NPM1 proteins are comprised of three different regions: the N-terminal domain (NTD), the central region, and the CTD, each with a distinct functional role (illustrated in Figure 2) [9]. The NTD is highly conserved and acts as a core domain due to its structural integrity and NES region. Post-translational phosphorylation of the NTD region of NPM1 results in instability and the induction of apoptosis but also limits the ability of cells to prevent DNA damage [10]. The oligomerisation of NPM1, on the other hand, causes cellular proliferation and localisation out of the nucleus to the cytoplasm by two weak NES regions [10]. NES interacts with a shuttling receptor XPO1, also known as Exportin 1 or CRM1 export protein (Chromosomal Maintenance 1), which subsequently causes XPO1 association with NES-containing proteins that result in cytoplasmic relocation of NPM1; this deviation gives rise to cytoplasmic NPM1 (cNPM1) [11,12]. In cases of NPM1 mutants, the additional NES at the CTD alongside two weak NES at the NTD results in strong associations with XPO1 and as a consequence, nuclear export to the cytoplasm dominates [3]. Studies have shown that cNPM1 impedes protein-protein interaction and subsequent nuclear interactions for example CCCTC-binding factor (CTCF) [13]. Through cNPM1 interactions, CTCF is relocalised to the cytoplasm, and consequently, gene regulation, RNA splicing, and DNA looping are reduced; abolishment of this interaction resolves CTCF DNA binding and nuclear concentrations [13].

The central region of NPM1 contains highly acidic amino acids (aspartic and glutamic acids) with their negative charge properties allowing mimicry of nucleic acids and thereby influencing the nuclear localisation signal (NLS). NLS regions interact with importin alpha and beta causing nuclear translocation of the NPM1 protein. The acidic central region of NPM1 facilitates histone binding to both core and linker histones for the formation of perinuclear heterochromatin [14]. Additionally, the central domain contains basic regions, with both influencing the ribonuclease activity of NPM1 and ribosome biogenesis activity [15]. Ribosomal RNA (rRNA) processing via NPM1’s endoribonuclease activity converts pre-rRNA to mature 28S rRNA.

The CTD contains predominantly basic and positively charged amino acids influencing its role in binding to nucleic acids. At the CTD, the nucleolar localisation signal (NoLS) domain allows the localisation of NPM1 to the nucleolus with aromatic residues (tryptophan 288 and 290) forming a three-helix structure and stabilises NoLS due to its hydrophobic core. The lack of aromatic residues is common in NPM1 mutant proteins and results in the unfolding of its helical structure which inhibits nuclear import and nucleolus import of NPM1. Aberrant localisation of NPM1 is typical amongst AML patients.

### 2.2. Functions of NPM1

#### 2.2.1. Ribosome Biogenesis

NPM1 is a multifunctional protein influencing processes involved in ribosome biogenesis, mRNA transcription, embryonic development, DNA repair, chromatin remodelling, and apoptosis. The most well-studied function of NPM1 is its involvement in directing ribosome biogenesis [16].

A mechanism known as liquid-liquid phase separation (LPS), has been proposed to explain the effects of NPM1 localisation in the nucleolus on ribosome biogenesis. Modulation of LPS occurs via homotypic and heterotypic interactions of the three key regions (NTD, central region, and CTD) of the NPM1 protein [17]. Three types of interactions are interplayed for the LPS mechanism to occur: (1) homotypic oligomerisation, (2) heterotypic interaction with rRNA, and (3) heterotypic interaction of R-motif-rich proteins.

The nucleolus subcomponent—the fibrillar centre (FC), is responsible for rRNA transcription. In the initiation of rRNA gene transcription, two components of the FC are formed: the dense fibrillar component (DFC) and the granular component (GC) [18]. The GC is dominated by homotypic interactions, which refer to NPM1-NPM1 interactions via electrostatic charges that result in the formation of a pentamer. Associations occur primarily through the NTDs and the intrinsically disordered regions (IDR) of NPM1 [19]. Heterotypic interactions with rRNA predominate at the DFC. The physicochemical properties of the nucleoplasm are altered, affecting surface tension and translocation out of the nucleolus [17,20]. Ribosomal proteins and pre-rRNA components (e.g., CDK1) interact with nucleolar scaffolds to allow for NPM1 sequestration and the formation of pre-ribosomal particles to be transported from the nucleolus to the nucleoplasm [21]. The most well-studied NPM1 interactions are the heterogenic interactions amongst R-motif-containing proteins. Direct interaction of ribosomal protein L5 (rpL5) with NPM1 allows shuttling and colocalisation of 5S rRNA, premature 60S rRNA, 80S ribosomes, and polysomes. Co-immunoprecipitation of NPM1 potential partner proteins such as RPL23, RPS7, RPL35, and RPS20 indicate its role as a scaffold in ribosome biogenesis within the nucleolus. NPM1 concentrations are particularly high in proliferating cells and were essential for ribosome maturation in the nucleolus [22].

#### 2.2.2. RNA Processing

DNA damage causes defects in mRNA processing, which leads to potential translation errors downstream; however, this is often remedied by translesion synthesis which is employed as a DNA repair mechanism by Polymerase Eta (Poly eta), a low-fidelity polymerase [23,24]. NPM1 is a key regulator of Poly eta by protecting it from degradation, as evidenced by the increased rate of Poly eta degradation in cells with higher mutant cytoplasmic NPM1 copies [24]. Therefore, translesion maintenance is important to consider when determining the prognosis of patients with *NPM1*-mutated AML.

A recent study also noted the impact of long noncoding RNA (lncRNA) increasing NPM1 mutant samples. Analysis using genome-wide RNA sequencing concluded that the introduction of lncRNA led to an increase in cell differentiation proteins; THSB1, MAFB, and ASB2. In addition, in vivo experimentation on mice confirmed decreased survival and accelerated AML pathogenesis in the presence of lncRNA. Decreased levels of lncRNA increases sensitivity to the chemotherapy drug, cytarabine, thus affecting treatment outcome [25]. Overall, an increase in cytoplasmic NPM1 mutants causes an increase in nuclear retention of lncRNA, ultimately contributing to the AML disease course [26].

#### 2.2.3. Chromatin Remodelling

Centrosome duplication influences the activation of chromatin and thus microtubule distribution. Correct chromatin remodelling allows a smooth transition of the cell cycle from interphase to metaphase [27]. The formation of CRM1/Exportin 1 contributes to the translocation of centrosomes and ribosomes with NES-bearing proteins. NPM1 contains two weak NES regions and interacts with the Ran-Crm1 complex. The formation of bipolar spindles requires regulation by the Ran-Crm1 network to ensure centrosome duplication [28,29]. Therefore, centrosome localisation to NPM1 is promoted by Ran-Crm1 complexes; the mutation T95D at the NES region of NPM1 inhibits centrosome duplication [30]. Histone chaperone activity is similarly affected by *NPM1* mutations. Assembly of the histone octamers, removal of DNA from histones, and transfer of histones from other chaperone proteins or enzymes are all roles of histone chaperones that are associated with NPM1 [31]. Collectively, the acidic and basic characteristics of the structural components of NPM1 allow the binding of nucleic acids and histones [32,33].

#### 2.2.4. Regulation of Apoptosis and Cellular Growth

Within healthy cells, NPM1 has the capacity to trigger either cell proliferation or apoptosis. However, a shift in homeostasis can affect NPM1 translocation to the cytoplasm, which in turn affects protein interactions in the cytoplasm profoundly. NPM1-induced activation of p53 stress-response mechanisms leads to a mitochondrial-associated release of extrinsic factors such as caspase and cytochrome c [34]. Mouse double minute 2 homolog (MDM2) is a negative regulator of p53, thus preventing its role as a tumour suppressor [35,36]. NPM1 directly binds to MDM2, hindering its inhibitory role on p53. Notably, this NPM1 mechanism indirectly affects p53 activity as indicated by studies on NPM1-negative mice and embryo cell lines that still express p53. The cysteine 275 to serine NPM1 mutant is incapable of undergoing S-glutathionylation, resulting in the release of nucleolar nucleic acids from NPM1. Consequently, nucleolar stress prevents translocation of NPM1 to the cytoplasm and binding to MDM2; this leads to the limited activation of p53 which is suggestive of nucleolar oxidation. The release of p14^ARF^ by NPM1 allows binding to MDM2 via rpL5. ARF-binding partners, such as MDM2 and NPM1, associate with ribosomal proteins, which implicates its role in p53 homeostasis [37]. Studies have also implicated direct interactions of NPM1 with p53 [38]. Therefore, NPM1 protein activity in apoptotic initiating pathways such as p53 are involved in both its roles in nucleolar stress and ribosome production [39]. Recent studies have also suggested that p53 mutated AML patients display distinct molecular signatures compared to p53 wild-type plus *NPM1* mutation-positive AML patients [40]. Therefore, the data strongly suggests that specific molecular pathways are involved between NPM1 and p53.

#### 2.2.5. Genome Instability

DNA repair response mechanisms are key to maintaining proper cell differentiation and genome stability. Post-translational modification of NPM1 regulates cell cycle proteins and promotes stability. DNA double-stranded lesions caused by UV results in the initiation of the nucleotide excision repair pathway (NER) which is overexpressed in *NPM1*-mutated AML, and this increases its activity as a chromatin-binding factor by upregulating proliferating cell nuclear antigen (PCNA), a NER protein. Subsequently, PCNA forms a homo trimer, interacts with cell cycle regulator p21 in the nucleus, and promotes DNA repair [32,41]. Release of retinoblastoma tumour suppressor (pRB) is also initiated by UV, causing dephosphorylation of NPM1 at Threonine 199, 234, and 237 by protein phosphatase 1 β (PP1β). Subsequently, pRB causes activation of E2F1-dependent DNA repair mechanisms [42]. NPM1-mutants, therefore, prevent fine tuning of DNA repair pathways by direct protein-protein interaction such as with apurinic/apyrimidinic endonuclease 1 (APE1) protein. Consequently, a break in equilibrium leads to an increase in DNA repair proteins translocation to the nucleus thus, cell proliferation is halted and normal ribosome biogenesis is impaired [43]. As mentioned above, the ARF-binding proteins also influence the endonuclease activity of NPM1, which in turn modulates the apoptotic and genome stability of cells [44,45].

## 3. *NPM1* Mutations in AML and Other Malignancies

According to the current WHO classification of hematopoietic malignancies, *NPM1*-mutated AML has been acknowledged as a distinct entity [46]. Nonetheless, it is usually accompanied by concomitant mutations, including but not limited to *FLT3*-ITD (FMS-like receptor tyrosine kinase-3 internal tandem duplication), *DNMT3A* (DNA methyltransferase 3 alpha), *IDH1/2* (isocitrate dehydrogenase ½), and *TET2* (Tet Methylcytosine Dioxygenase), raising speculation of synergistic interactions that promote AML transformation and progression [3,47,48,49]. Indeed, mutated *NPM1* is a driver mutation and often a secondary event to pre-existing mutations generated by clonal hematopoiesis of indeterminate potential (CHIP) [2,7,47,50]. The most recognized risk stratification system for AML is the European LeukemiaNet (ELN) genetic risk classification at diagnosis. The ELN risk classification states that cytogenetically normal *NPM1*-mutated AML, without *FLT3*-ITD, belongs to a favorable risk group. Cytogenetically normal *NPM1*-mutated AML with *FLT3*-ITD belongs to an intermediate risk group exhibiting adverse outcomes, especially in the presence of the aforementioned co-mutations [51]. It must be noted that 85% of *NPM1*-mutated AML cases exhibit normal karyotypes, with the remaining cases showing various cytogenetic abnormalities e.g., del (9q), +21, +8, and +4 in AML [52].

*NPM1* insertional mutations have also been found in myeloid neoplasms including myelodysplastic syndrome (MDS), myeloproliferative neoplasm (MPN), and chronic myelomonocytic leukaemia (CMML). In lymphoid neoplasms, translocation events are reported. Accounting for about 2% of *NPM1*-mutant-related diseases, *NPM1*-mutation in MDS has been associated with a higher risk of secondary AML and is most commonly found in MDS with an excess blast (MDS-EB) [53,54]. Molecular diagnosis of MPN occurs from the presence of mutant *JAK2-V617F* and associated diseases include essential thrombocythemia (ET) and myelofibrosis (MF) of which *NPM1*-mutations have been found on a rare occasion [55,56]. This shows the high sensitivity of *NPM1*-mutant cells seen in MPN and possible regimens without the need for stem cell transplantation. Contributing to 3% of *NPM1* mutant cases, CMML with *NPM1* mutation shows chemosensitivity to anthracyclines and cytarabine (7 + 3 induction chemotherapy), and hypomethylating drugs have promising effects in maintaining remission [55,57]. On the other hand, for translocation events of NPM1, treatment has been associated with the elimination of the fusion protein directly. This includes anaplastic large-cell lymphoma with NPM1-ALK (anaplastic lymphoma kinase) and T-cell lymphoma with NPM1-TYK2 (tyrosine kinase 2) [58,59]. It must be noted that only a small proportion of patients who exhibit *NPM1* mutations outside of AML can be found.

## 4. Measurable-residual monitoring in *NPM1*-mutated AML

With the advent of modern technology and improved molecular techniques, MRD has become indispensable for AML patient management and monitoring of mutant NPM1 before, after, and during the course of treatment [3,60,61,62,63]. MRD positivity has been defined by the ELN as either the conversion of CR_MRD−_ to CR_MRD+_, or the increase of PB/BM-MRD by ≥1log_10_ between two positive samples [60]. For better harmonization of *NPM1*-mutated AML data, log reduction of MRD should be the preferred choice for reporting, as multiple studies have shown that MRD kinetics are reliable prognostic indicators of the outcome, but not absolute MRD levels [62,64]. Technical aspects and sample collection for MRD monitoring are extensively stated in the ELN guideline [51].

### 4.1. Conventional Technologies for MRD

Conventional technologies for MRD detection include flow cytometry and molecular MRD assessments by qPCR and ddPCR. In AML patients receiving hematopoietic stem cell transplantation (HSCT), MRD positivity at pre-HSCT by flow cytometry is strongly correlated with worsened overall survival and leukemia-free survival (LFS) than MRD-negative patients. Pre-HSCT MRD negativity is also associated with a reduced incidence of relapse supporting the predictive significance of MRD monitoring on survival and post-HSCT relapse [65]. Similarly, in high-risk AML patients with normal karyotype, flow cytometric MRD positivity is strongly associated with relapse-free survival in these patients [66].

When evaluating MRD using multiparametric flow cytometry specifically in *NPM1*-mutated patients, a unique immunophenotype of AML patients was identified [67]. Leukaemic cells in patients with type A *NPM1* mutation strongly expressed myeloperoxidase (MPO) and CD33 with dim expression of other myelomonocytic antigens commonly expressed in AML including CD13, CD65, CD15, and CD14. Its utility in the MRD monitoring of *NPM1*-muated AML in general is yet to be confirmed. Therefore, molecular MRD strategies targeting these specific mutations are crucial for more specific analyses of MRD kinetics and clonality.

As per the latest MRD guidelines from the ELN, molecularly defined groups such as *NPM1*-mutated AML should be monitored by qPCR and ddPCR; the implication of both approaches have been extensively published and reviewed [2,6,60,61,68]. Patients that fail to achieve a 4-log reduction of MRD have a significantly higher risk of relapse as well as shorter survival [64]. *NPM1* mutations are commonly monitored using RNA normalized to *ABL* transcripts, a technique that is used to monitor other fusion genes in a similar fashion. Meanwhile, recent studies and in-house data (data not published) have shown that quantification of *NPM1* mutation allele frequency at the DNA level is a good alternative to RNA-based assay. In spite of lower sensitivity than RNA-based assays, clinical correlation with DNA mutation allele frequencies are also observed. This permits flexibility of the MRD protocol and alleviates the need to normalize to *ABL1* which has a significantly slower expression rate than *NPM1* [68,69].

### 4.2. Advanced Technologies for NPM1 MRD Monitoring

Application of NGS for MRD monitoring can be conducted at bulk tumour level and single-cell level, although none of them are routinely applied. Bulk tumour sequencing is most commonly adopted at the early stages of AML as screening tools that allow simultaneous screening of a vast number of mutations. Whole exome sequencing (WES) can detect *NPM1* mutations months prior to the onset of de novo AML even though coverage is generally lower than gene panel sequencing [70]. NGS also permits the sub-classification of *NPM1* mutations as non-type-A mutations that are reported to confer shorter survivals and inferior clinical outcomes than wild-type or type A mutations [71,72,73]. Despite the demanding nature of NGS bioinformatic analyse and interpretation, its application in MRD detection is beneficial especially when most PCR-based assays detect *NPM1* type A mutants but not other *NPM1* mutation subtypes.

Similarly, the application of single-cell technology is continuously under development for MRD monitoring for improved resolution. Both DNA and conventional RNA single-cell sequencing have been employed in MRD monitoring in AML but are not routinely used due to cost and the high requirement for sample preparation [74,75]. With the inclusion of surface marker staining, single-cell multi-omic analysis further increases the resolution of the MRD in *NPM1* mutated AML patients [76]. In these studies, clonal heterogeneity of the *NPM1* mutant clone has been revealed, and serial monitoring has shown that the population changes dynamically with the acquisition of *NPM1* and *FLT3* mutations during the intermediate stages of the disease. Clonal switch of *NPM1* co-mutation with *PTPN11* to *WT1* is also resolvable at relapse, demonstrating the benefits of high resolution NGS. The ultra-sensitivity of single-cell technology is demonstrated with <1% of mutant cells carrying *NPM1* or other SNPs out of as few as 7000 cells being sequenced per time point [74]. When single-cell analyses are coupled with mass cytometry and further supplemented by bulk tumour sequencing, dynamic monitoring of AML responses to chemotherapy can be achieved [77]. In a cohort of 32 AML patients, 36-plex mass cytometry has been used to monitor 24 h responses to standard 7+3 induction chemotherapy. The employment of machine learning analysis of the cell population has further identified high p-ERK1/2 in blast cells at 24 h being a predictive marker for poorer MRD and five year survival. While the majority of the p-ERK1/2^low^ patient achieved CR, the MRD positivity observed in a few p-ERK1/2^low^ patients is due to the presence of *NPM1* mutations. This provides a novel method that deciphers the effect of intracellular signalling on MRD dynamics [77]. This also envisions the significance of early evaluation of MRD adopting a multi-omic approach.

## 5. Targeting *NPM1*-Mutated AML

**Figure 3:**
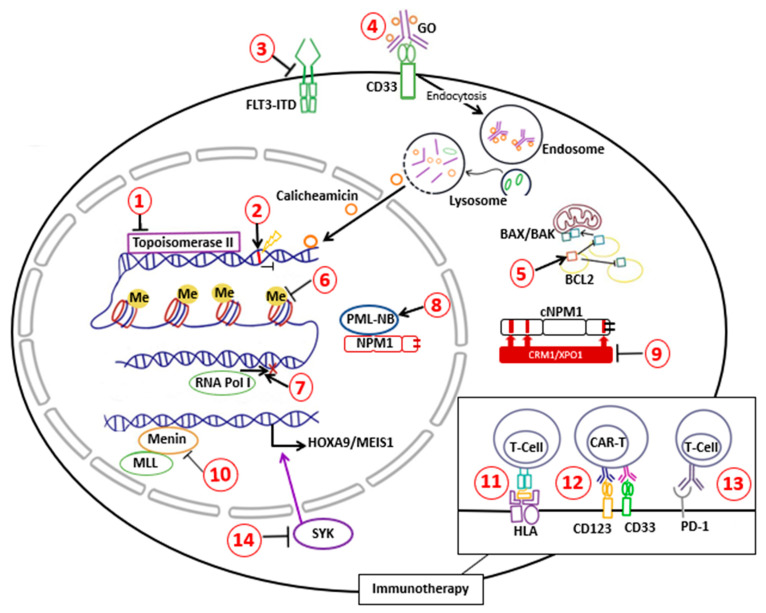
Targeted therapy for *NPM1*-mutated AML. (**1**) Anthracycline, (**2**) Cytarabine, (**3**) FLT3 inhibitors, (**4**) Gemtuzumab ozogamicin (GO), (**5**) Venetoclax, (**6**) Hypomethylating agents (Azacitidine), (**7**) Actinomycin D/Dactinomycin, (**8**) Arsenic trioxide and ATRA, (**9**) XPO1 inhibitors (Leptomycin) with cytoplasmic NPM1 (cNPM1), (**10**) Menin inhibitors disruption of Menin with histone methyltransferase MLL1 (KMT2A), Immunotherapy located on cell membrane: (**11**) HLA-dependent T cell immunotherapy, (**12**) CAR-T immunotherapy, (**13**) Anti-PD-1 immunotherapy, and (**14**) SYK inhibitor.

At least one-third of AML cases are *NPM1*-mutated, so developing treatment approaches for this population is becoming crucial. Upon initiation of treatment, a risk assessment is recommended by the ELN for diagnosis and management [51]. Primary determinants of the therapeutic approach are age, comorbidities, and diagnostic molecular profile [78]. Standard treatment protocol for young and fit *NPM1*-mutated AML patients involves induction with intensive chemotherapy followed by consolidation chemotherapy. Co-occurrence of *FLT3*-ITD and/or *DNMT3A* mutation is found in approximately 66% of *NPM1*-mutated AML, which portends an inferior prognosis [79,80,81,82,83], constituting an indication for allogeneic haematopoietic stem cell transplantation (HSCT) in eligible patients. The persistence of *NPM1* mutant transcripts following induction or consolidation chemotherapy is associated with high relapse rates [64,84]. Patients with the persistence of *NPM1*-mutant transcripts in the peripheral blood after the second cycle of chemotherapy were associated with a higher risk of relapse compared to those with absent *NPM1* mutant transcripts (82% *versus* 30%) after three years of follow-up [85]. In the United Kingdom National Cancer Research Institute (NCRI), an AML17 study that involved 107 patients with *NPM1*-mutated AML receiving allogeneic HSCT, the estimated two year overall survival was 83%, 63%, and 13% in patients with negative, low (<200 copies per 10^5^ ABL in the peripheral blood and <1000 copies in the bone marrow aspirate), and high levels of MRD respectively after a median follow-up of 4.9 years [86]. In patients who were FLT3-ITD positive with low-level MRD pre-HSCT, the two year overall survival was only 17% [86]. In *NPM1*-mutated AML patients undergoing allogeneic HSCT, positive measurable residual disease (MRD) prior to transplantation was associated with high risk of post-HSCT relapse [86]. The one year relapse-free survival post HSCT in patients with positive MRD pre-HSCT is 45% [86]. In another study, the two year cumulative risk of relapse was 65% in patients who were MRD-positive before allogeneic HSCT [87]. On the other end of the spectrum, patients who are unfit or over 75 years of age are given Venotoclax with either HMA or low dose cytarabine (LDAC). As a result of the variety of treatments available, the current review will outline current and novel agents, as well as the mechanisms involved in targeting *NPM1*-mutated AML (Figure 3).

### 5.1. Current Agents or Standard Treatment

#### 5.1.1. Anthracycline

*NPM1*-mutated AML patients are commonly treated with the “7 + 3” regimen which is a combination of cytarabine for 7 days and anthracycline for 3 days. The favourable response of “7 + 3” has enabled it to become the standard of care for AML due to its CR rate of 60–80% in younger patients and 40–60% in older adults (>60 years) [88,89]. Anthracyclines are a group of potent chemotherapeutics that lack selectivity due to their aromatic ring and attached amino-sugar, thus may warrant unwanted side effects such as cardiac abnormalities in the elderly AML population [90,91]. The chemical structure allows for the transfer of electrons between structures, causing an increase in free radical formation that results in the peroxidation of lipids within the cell [92]. The most clinically used anthracyclines include Daunorubicin (daunomycin), Doxorubicin, Epirubicin, and Idarubicin. Various mechanisms have been suggested, one being a DNA intercalator. The minor groove of DNA has been found to interact directly with Daunorubicin at sequence CGTACG to form a “daunorubicin-d(CGTACG)” complex [93]. A stable structure determined by X-ray crystallography shows the complex involves water molecules, anthracyclines, and DNA forming hydrogen bonds [93]. Next, doxorubicin has been shown to cause double-strand breaks (DSB) by the inhibition of DNA topoisomerases II [94,95]. During replication, normal DNA topoisomerase II prevents DSB by performing DNA catenation and knotting. Anthracyclines prevent the formation of the intermediate DNA-Topoisomerase II cleavage complex (Top2cc), resulting in apoptosis due to a DSB in the DNA [96]. However, it should be emphasised that due to the high potency of anthracyclines, cardiotoxicity is frequently observed and likely attribute to chromatin damage by histone removal [95], which is believed to cause apoptosis or necrotic events.

Another mechanism of anthracyclines is via the immunostimulatory pathway STING, which is a common immune response pathway upon pathogen entry [97]. STING modulates the release of dead-cell antigens contributing to the cascade of immunogenic cell death [98]. Caspases and ATP are released allowing stress-like signals to initiate an immune response. Innate immunity is manifested in cells through the activation of interferon regulatory factors (IRFs), with a focus on IRF1 genes and IRF3 phosphorylation [99]. IRF3 has been shown to contribute to the transcription of type I interferons and is linked via the activation of toll-like receptor (TLR)-3 and TLR-4 signalling [100]. Thus, the activation of the STING pathway, which causes IFN upregulation, is a key mechanism of anthracycline that activates immune responses [101,102].

#### 5.1.2. Cytarabine

Cytarabine is used with anthracycline (7 + 3 regimen) during the remission induction. High-dose cytarabine (HDAC) is used in the consolidation of AML. LDAC is prescribed for patients unfit for intensive induction chemotherapy. Cytarabine (arabinosylcytosine, ARA-C) is a pyrimidine analogue that competes with the base pair cytidine in both DNA and RNA. Therefore, by limiting DNA polymerase beta and base excision repair, inhibition of DNA replication occurs, particularly at the S phase [103,104]. In 2014, CPX-351, a new encapsulated drug with a 5:1 ratio of cytarabine and daunorubicin was run in parallel with the standard treatment protocol (7 + 3 regimen) in newly diagnosed elderly *NPM1*-mutated AML individuals. Notably, older adults showed longer remission rates in the CPX-351 arm and had a similar safety profile as the intensive standard chemotherapy arm [105]. This suggests the significance of cytarabine and the appropriate isoform of anthracycline to be administered to both the elderly and the young. In *NPM1*-mutated AML patients, cytarabine efficacy is comparable to anthracycline efficacy due to cytarabine’s role in inhibiting DNA replication and the capacity of *NPM1* to regulate transcriptional processes. As a result, it is highly effective against rapidly diving cells regardless of the subtype of mutated *NPM1* [106,107].

#### 5.1.3. FLT3 Inhibitors

As a large proportion (about 25%) of *NPM1*-mutated patients carry *FLT3* mutations, initial screening of patients with *FLT3*-ITD or FLT3-TKD (tyrosine kinase domain with missense point mutation) benefit greatly from FLT3 inhibitors in combination with various chemotherapy [78]. Under normal circumstances, FLT3 receptor activation occurs upon binding of FLT3 ligand and the subsequent rotational change of amino acids—aspartate, phenylalanine, and glycine into an active conformation, which causes dimerization and tyrosine autophosphorylation [108]. Subsequently, TKD is activated and initiates a signalling cascade of pathways—JAK/STAT, RAS/MAPK, and PI3/AKT/mTOR signalling [109,110]. The most commonly used and researched FLT3 inhibitor, first-generation Midostaurin, has the capability of targeting a wide range of receptor tyrosine kinases (RTK), including FLT3 [111,112].

*FLT3*-ITD mutants exhibit persistent activation in the absence of ligand binding, resulting in the formation of its dimeric form and downstream cell proliferation [113]. Midostaurin acts by binding on the ATP binding pocket of FLT3, preventing activation and autophosphorylation [114]. Second-generation FLT3 inhibitors, Gilteritinib and quizartinib, are commonly used for relapsed/refractory *FLT3*-ITD positive AML cases [115]. Collected from in vitro analysis, Midostaurin shows possible off-target inhibition on platelet-derived growth factor receptors (PDGFRs), cyclin-dependent kinase 1, spleen tyrosine kinase, and vascular endothelial growth factor receptor [116]. Second-generation FLT3 inhibitors have greater specificity for FLT3 but lack inhibition of other RTK mechanisms, which may help to amplify anti-leukemic effects. However, because of the lack of specificity, first-generation FLT-3 inhibitors are both more potent and toxic, thus unsuitable for the elderly and unfit. In addition, it must be noted, recently the acknowledgment of *FLT3*-ITD-positive MRD pre-transplant has been associated with higher rates of relapse. A recent study has shown better overall survival within double positive *FLT3*-ITD and NPM1 mutations in both younger and older patients, signifying *NPM1*’s influence on using *FLT3*-ITD inhibitors [117]. Therefore, studies relating the efficacy of FLT3 inhibitors still require further investigation of the potential risk of how *FLT3*-ITD mutants play in NPM1-mutated AML patients. Some studies also show combination with venetoclax show molecular clearance of *FLT3* mutations [118].

#### 5.1.4. Gemtuzumab Ozogamicin

The CD33 antibody-drug conjugate (ADC)—gemtuzumab ozogamicin (GO (CMA-676); Wyeth Laboratories, Mylotarg^®^, Philadelphia, PA, USA) is the next FDA approved drug for AML. According to the national comprehensive cancer network (NCCN), GO administration should be given to either CD33-positive patients as post-remission therapy or patients placed in the favourable and intermediate cytogenetic risk groups [119]. In combination with intensive induction chemotherapy, GO has been shown effective in *NPM1*-mutated AML. In terms of NPM1 influence on GO treatment effect, CD33 cells are abundant among NPM1-mutated AML patients [120]. Several trials involving the antibody conjugate approach conclude that standard intensive chemotherapy compared with the addition of GO leads to a reduction in relapse and a better benefit/risk ratio [119,121,122]. In addition, a prospective study (AMLSG 09-09 Phase III trial) showed benefits in patients less than 70 years old with *NPM1*-mutated/*FLT3*-WT genotype [123].

GO is useful due to its conjugated cytotoxic antibiotics, calicheamicin. Calicheamicin (N-acetyl-γ-calicheamicin 1,2-dimethyl hydrazine dichloride) inflicts double-stranded cleavages on DNA which result in apoptosis [124]. The CD33 IgG4 antibody prompts action against CD33 antigen-presenting leukemic myeloblasts due to its specificity. The use of IgG4 is critical because (1) its chemical structure allows a longer half-life amongst its isoforms, and (2) a poor Fc region limits the initiation of antibody-mediated cytotoxicity and thus improves the safety profile as an antibody-drug conjugate [125]. CD33 expression occurs at all stages of myeloid differentiation but is particularly abundant in *NPM1*-mutated AML [120]. Calicheamicin is internalized when IgG4 binds to CD33, causing endocytosis and lysosome entry into the endosome. As a result of lysosome C release, the reduction of pH from 7.4 to 4 causes hydrolysis of covalently AcBut linker-bound calicheamicin to be released [126]. Next, glutathione reduction produces active forms of the organic compound enediyne, allowing it to directly bind to the minor groove in DNA, causing double-strand breaks [124].

#### 5.1.5. Venetoclax

The standard treatment protocol for newly diagnosed *NPM1*-mutated AML patients who are unfit and/or elderly (>60 years), is a combination of venetoclax and HMA or LDAC. The small FDA approved molecule, venetoclax, targets the NPM1 apoptotic pathway by inhibiting B-cell leukaemia/lymphoma-2 (BCL-2). BCL-2 is a key anti-apoptotic protein that sequesters BAX/BAK, thereby regulating mitochondrial outer membrane permeabilization (MOMP) [127]. BAX/BAK are key regulators of apoptosis which shuttle out of the cytosol in response to stress, initiating MOMP and causing cell death. Release of BAX/BAK occurs via binding of BCL-2 homology 3 (BH3) containing proteins (such as apoptotic BIM, PUMA, and BID) to BCL-2 [128,129]. Venetoclax inhibits BCL-2’s ability to bind to BAK/BAX as a “BH3-mimetic” because of its structural similarity to a BH3-containing protein, resulting in competitive inhibition [130]. Studies on the human monocytic leukaemia cell line THP-1 with *NPM1* mutation reveal an increase in sensitivity to chemotherapy drugs through suppressing NF-κB activity and BCL-2/BAX expression, which is pertinent to the efficacy of venetoclax seen in *NPM1*-mutated AML [131]. Predictive markers for venetoclax sensitivity show that relapsed/refractory AML or untreated AML patients exhibiting high BCL2 protein concentrations show better outcomes [132]. However, this is not the case in all patients. A possible reason is the presence of *FLT3*-ITD or loss of *TP53* causing a mitochondrial defect in *NPM1*-mutated AML patients. Additionally, *IDH1*/2 mutations occur in *NPM1*-mutated AML with increasing age influencing the efficacy of venetoclax-based therapy [133].

#### 5.1.6. Hypomethylating Drugs

The use of HMA drugs in standard protocols is due to their proven efficacy and effect in MDS patients, but it must be noted that AML contains specific clinical characteristics, making it distinct from MDS. Mechanistically, the influence of NPM1 on DNA methylation was studied in mouse models with mutant DNA methyltransferase (*DNMT3A*) and *NPM1. DNMT3A* mutants in hematopoietic stem cells (HSC) and commonly associated as a concomitant gene for *NPM1*-mutated AML, and precursors for de novo adult AML cases. Cooperation between the two genes shows the activation of promoters influencing preleukaemic mechanisms such as PI3K/Akt/mTOR signalling, stem cell pathways, and HSC cycling. Thus, showing the significance of using demethylating drugs such as azacytidine and decitabine in treating *NPM1*-mutated AML [134]. Azacitidine prevents abnormal growth of cells by targeting DNMT1 causing a reorganisation in the chromatin structure. It can be easily transported due to its similarities to nucleotide cytidine and can be easily incorporated into DNA both RNA. Classified as an antimetabolite and a demethylating agent, trials developed till phase 3, QUAZAR AML-001 trial (NCT01757535), show good tolerability and outcome in AML, therefore suggesting a good alternative for elderly patients who are unfit for intensive chemotherapy [135]. HMA’s have also been used in relapsed/refractory patients due to their ability to sensitise leukemic cells for further chemotherapy and its utility in maintenance therapy.

#### 5.1.7. Actinomycin D

Another potential target is inducing nucleolar stress on *NPM1*-mutated cells. Actinomycin D (or dactinomycin) is a low-cost chemotherapy drug that induces nucleolar stress by targeting RNA polymerase I to inhibit transcription elongation. The structure of Actinomycin D resembles the ring structure of nitrogenous bases in DNA, therefore enabling its intercalation into DNA. At low doses, actinomycin D induces stress responses in the cell by increasing ROS through the activation of cyclic GMP-AMP synthase signalling and the release of mitochondrial DNA [136]. Mechanistically, NPM1-mutants are involved in altering the normal activity of mitochondrial biogenesis, and the lack of PML-NB in NPM1-mutant AML prevents TP53 activation. [136]. Thereby, a change in NPM1-mutants will alter ROS production via the mitochondria. Thus, actinomycin D is an effective treatment for the elderly in modulating defective mitochondrial function and has been used in combination with venetoclax to potentiate mitochondrial-induced apoptosis [137].

### 5.2. Novel Agents and Future Directions

#### 5.2.1. Arsenic Trioxide (ATO) and All-Trans Retinoic Acid (ATRA)

ATO and ATRA are popular molecular targeted treatment strategies that inhibit downstream phosphorylation pathways and have been implicated in acute promyelocytic leukaemia (APL) treatment. The myeloid leukaemia cell differentiation protein (Mcl-1) inhibits apoptosis by promoting the growth of AML cells. Studies have suggested ATO inhibits AKT and the extracellular signal-regulated kinase 1/2 (ERK1/2) cascade thereby upregulating Mcl-1. Usually, Mcl-1 is downregulated via the AKT (protein kinase B) pathway by GSK-3β inhibiting Mcl-1 phosphorylated at Ser159 [138]. In addition, ERK1/2 inhibits Mcl-1’s phosphorylation at Thr163. The phosphorylation at both regions on Mcl-1 triggers the release of more caspases and starts the destruction of AML cells [138]. With the use of venetoclax to downregulate Mcl-1, clinical trials show the synergistic effects of ATO to promote venetoclax activity [138].

As a curative frontline treatment for APL, ATO-ATRA molecularly targets the oncogenic fusion protein; promyelocytic leukaemia–retinoic acid receptor α (PML::RARA) [139]. By applying the knowledge gained from APL treatment which relies on the degradation of oncogenic PML-RARA, targeting the mutated NPM1 protein is an obvious choice of treatment. In APL, ATO targets proteasomal degradation via PML-NB formation and SUMO-1 association; these results were similar to those observed when using ATO on *NPM1*-mutated AML cells [140]. ATO possesses oxidative abilities via the ROS-mediated p53 pathway activation of NPM1, to which *NPM1*-mutated AML cells with cysteine 288 are highly sensitive [141]. Arsenic acts as a pro-oxidant drug that induces apoptosis and anti-leukemic effects. With regards to ATRA on *NPM1*-mutated AML, a possible explanation for its efficacy is its synergy with ATO as seen in both APL and AML cases. Notably, daunorubicin sensitivity increases *NPM1*-mutated AML cells’ post-treatment sensitivity to ATO-ATRA, showing promise as possible sensitisers for leukemic cells before chemotherapy [141].

#### 5.2.2. XPO1 Inhibitors

XPO1 is a key protein that interacts with the NES regions of NPM1, resulting in an increase of cytoplasmic NPM1 proteins due to increased NES signalling. Therefore, targeting chaperone proteins of NPM1 will limit its activity in the cytoplasm, thereby sequestering it to the nucleolus/nucleus. Examples of XPO1 inhibitors include leptomycin, selinexor, and eltanexor. Leptomycin B has shown severe toxicity in vitro as it binds irreversibly to XPO1, therefore selinexor monotherapy was implemented in NPM1-mutated AML patients; however, selinexor has shown only temporary and insignificant clinical benefits due to its high toxicity [142]. The knowledge gained on the mechanisms of both leptomycin B and selinexor led to the development of second-generation eltanexor. It has significantly longer-lasting beneficial effects with prolonged survival and terminal AML differentiation due to a downregulation of HOX as seen in leukemic mice. Mechanistically, XPO1 inhibition decreases cNPM1, allowing an increase in NPM1 proteins within the nucleolus for its roles in maintaining ribosome biogenesis [142]. An advantage of eltanexor is its better tolerability amongst patients, allowing an increased dosing schedule to induce a greater anti-leukemic effect [143].

#### 5.2.3. Menin Inhibitors

Menin is a scaffold protein involved in many protein-protein interactions and most notably, it can act as an oncogenic cofactor when bound to KMT2A (histone-lysine-N-methlytransferase 2A (MLL1)) complexes, resulting in gene transactivation [144]. KMT2A is an important transcription factor that facilitates histone H3K4 trimethylation [145]. The involvement of NPM1 mutants in histone modification occurs by indirect interaction of the MLL1-Menin complex which directly binds to chromatin targets [146,147]. In *NPM1*-mutant AML cases, overexpression of HOXA and HOXB genes and cofactor MEIS1 are often observed. Inhibition of MLL-Menin complex binding results in the downregulation of HOX cluster genes, specifically HOXA9 (leukemogenic homeobox A9) and MEIS1 (myeloid stem progenitor homeobox transcription factor) [145]. This HOX/MEIS1 complex allows the recruitment of MLL3/MLL4 and CEBPα to further promote leukemogenesis. Recent studies show NPM1-CRM1 complexes are guided by distinct binding motifs but vary between NPM1 subsets and cell types (OCI-AML3 and 293T). Suggesting multiple pathways influencing the transcriptional effect of NPM1 on histone binding and contributing to specificity towards active chromatin binding [146]. As proven, one pathway involves HOXA and HOXB cluster genes, thus implying menin inhibitors have a potential indirect influence on histone binding.

Currently, a few drugs are undergoing early-phase clinical trials on relapsed/refractory AML patients with KMT2Ar/NPM1 to evaluate efficacy as either an MLL-Menin complex inhibitor (KO-539 (NCT04067336), SNDX-5613 (NCT04065399), and JNJ-75276617 (NCT04811560)) or menin inhibitor (DS-1594b (NCT04752163) and BMF-219 (NCT05153330)). However, due to the targeted nature of therapy, the careful identification of biomarkers indicating patient suitability for the use of menin inhibitors would be beneficial and a key strategy in overcoming unwanted leukemic activity. The clinical trial, SNDX-5613 (NCT05326516), is currently underway to evaluate the potential of using menin inhibitors with chemotherapy drug cocktails [148].

#### 5.2.4. HLA-Dependent T-Cell Immunotherapy

Another targeted-therapy approach on the rise is cancer immunotherapy treatments. As mentioned previously, NPM1 is a highly specific and stable mutation with relapses usually detected by increasing MRD [149]. Due to the nature of *NPM1*-mutated AML, with the driving factor being an oncoprotein, immunotherapy is an ideal approach that focuses on either targeting mutant NPM1-derived neoantigens or genetically engineering T cells specific for NPM1-mutated peptides. Various studies have attempted to determine ideal NPM1-derived immunogenic peptides suitable for HLA- (Human leukocyte antigen) induced T cell response. Efficient HLA presentation has been seen targeting 11 residues of the NPM1-mutated C-terminus due to the distinct amino acid sequence not normally found in human tissues. Specifically, HLA class I ligandomes: AIQDLCLAV, AIQDLCVAV, AVEEVSLRK 9-mer, CLAVEEVSL, LAVEEVSLR, and CLAVEEVSLRK have been studied [149,150,151,152]. Single-cell RNA sequencing has exhibited upregulation of immune responses and major metabolic pathways caused by increased cytokine-induced memory-like NK cells; the pathways activated relate to mitochondrial maintenance and show similar activity upon IL-15 activation of NK cells. However, it must be noted that peptide-HLA complex recognition is successful only in cells that express the target neoantigen. Occasionally, immuno-editing can occur, whereby the immune system constrains and promotes cancer growth in an attempt to control immune escape mechanisms; this is more common in cells with a higher mutational load.

#### 5.2.5. CAR-T Therapy

Another immunotherapy approach targeting NPM1 is the use of chimeric-antigen receptor T cells (CAR-T) and TCR-engineered T cell therapies. The most successful antibody therapy is the monoclonal antibody, GO, which shows promise in targeting the myeloid-specific transmembrane CD33. From this knowledge, bispecific CAR-T cells were designed that targeted CD33 as well as CD123, an IL3 receptor found overexpressed in leukemic neoplasms for example AML. Dual CAR targeting the CD33 and CD123 allows specificity to AML leukemic cells; this strategy is effective as a single expression of CD33 or CD123 by healthy cells will result in no or attenuated CAR-T cell response. The anti-CD123 first-generation CAR is attached to a CD3ζ domain, whilst anti-CD33 contains only CD28 and 41BB costimulatory domains [153].

#### 5.2.6. Immune Checkpoint Inhibition

Another T-cell immune response strategy involves targeting the programmed death 1 antibody (PD-1). PD-1/PD-L1-directed immunotherapy shows potential due to its expression in *NPM1*-mutated patients and absence in *NPM1*-WT. The ligand PD-L1, which inhibits the activation of T cells via PD-1 receptors downstream, is commonly overexpressed in tumour cells and is frequently associated with poor clinical outcomes [154]. Nivolumab, an anti-PD-1 antibody, is one example. It inhibits the immune checkpoint PD-1, which stimulates leukaemia-associated antigen (LAA) activity in both mutant and WT cytotoxic T lymphocytes but only in the presence of the NPM1 mutant peptide proliferation [155]. Nivolumab, in particular, targets leukemic progenitor/stem cells (LPC/LSC), a population thought to be responsible for AML relapse [155]. Studies show mixed conclusions, some suggest that PD-L1 expression does not directly influence T cell activation, while others show that inhibiting PD-1 signalling causes Treg expansion to be delayed. Anti-PD-L1 antibody limits Treg cell expansion in murine AML models, limiting disease progression [154]. According to the research, AML patients with higher levels of PD-1+ Treg cells have a better prognosis when undergoing anti-PD-1 medication. Therefore, due to the heterogeneity of AML patients, this new selective targeted immunotherapy may be advantageous for the prevention of relapse in PD-1^+^/NPM1^mut^ patient subgroups.

#### 5.2.7. SYK Inhibitor

Another target of interest is non-receptor spleen tyrosine kinase (SYK). Under normal circumstances, Lyn (tyrosine kinase protein) auto-phosphorylates Syk at Tyr518 and generates binding sites for the BCR signalling pathway regulators CBL, VAV1, and phospholipase C-gamma [156]. SYK inhibitors for *NPM1*-mutated AML are currently undergoing clinical trials, and these include entospletinib (GS-9973) and mivavotinib (TAK-659). The second-generation SYK inhibitor, entospletinib, is a reversible competitive small molecule with higher sensitivity to JAK-2, c-KIT, FLT 3, and RET [157]. Clinical trials (NCT05020665) on entospletinib for *NPM1*-mutated AML patients are still ongoing. Mivavotinib, a heteroaromatic SYK inhibitor, inhibits both SYK and FLT3 by competitively binding at the adenosine triphosphate-binding site. To note, AML patients with *HOXA9*/*MEIS1* overexpression show improved survival with entospletinib, suggesting its involvement with menin-MLL complex inhibition [158]. In vitro AML cell studies have shown that SYK inhibition perturbated AML growth via pathways similar to FLT3 pathway inhibition. Demonstrated in vivo, SYK interacts with and activates FLT3 directly, making *FLT3*-ITD more sensitive to SYK inhibition, further supporting the screening of *FLT3*-ITD as an important determinant of prognosis in AML patients [159]. Thus, clinical trials conclude that combination therapy with standard chemotherapy drugs (i.e., cytarabine and daunorubicin) show a better therapeutic response for *NPM1*-mutant AML with a better-predicted response rate for *FLT3*-ITD positive patients [158].

#### 5.2.8. Stress Inducing AML

Recent studies show the significance of stress in the promotion of AML due to the accumulation of IL-1β. One possible mechanism is via the activation of the NLR family pyrin domain containing 3 (NLRP3) inflammasomes leading to the progression of AML cells. Studies conducted on chronically stressed mice showed upregulation of *NLRP3* mRNA and further downstream analysis on AML cell lines saw the presence of high mobility group box 1 (HMGB1) protein. This suggests the influence of stress on the HMGB1-NLRP3 axis and is a potential target for preventing AML progression [160]. In addition, as previously noted, the cooperation between mutated *NPM1* and *DNMT3A* shows an increased expression of HMGB1, similar to what is hypothesised on mice exhibiting chronic stress. Thus, showing an overlap between the various suggested concepts. Another suggested mechanism for targeted therapy involves diverting immune escape by preventing the release of the immune suppressor protein galectin-9, which limits the release of cytokines. The stress-related hormone cortisol triggers the upregulation of neuronal receptor latrophilin 1 (LPHN1), and the upregulation of fibronectin leucine-rich transmembrane protein 3 (FLRT3). Subsequently, the release of galectin-9 causes immune escape [161]. With regards to stress management of AML, new studies show targeting stress-induced inflammation signalling pathways can further sustain current cancer treatment strategies. This includes pharmacological and psychosocial interventions preventing the upregulation of noradrenaline, adrenaline, and glucocorticoids showing potential in decreasing the overall survival of cancers such as non-small cell lung cancer [162,163]. Though further studies still need to be conducted on the effects of stress on haematological malignancies.

Altogether, it is evident that the genetic diversity of *NPM1*-mutated AML should be examined in terms of its mutational landscape and clonal evolution for better patient-specific treatment needs. Likewise understanding the immunophenotypic markers (e.g., CD4, CD34, and CD56) of patients can better understand immune resistance and escape mechanisms in AML [164,165]. One possible non-conventional strategy is meditation, which has been linked to 68 genes modulating interferon signalling [166]. With continued understanding of the clinical implications of stress and the immune system, modulation could support current AML treatment options to bolster LFS and overall survival.

## 6. Conclusions

Targeted therapy has become an important treatment strategy in targeting *NPM1*-mutated AML. Research focusing on understanding the functional biology of mutated *NPM1* showed numerous mechanisms contributing to disease progression. However, the underlying molecular pathways driving *NPM1*-mutated AML pathogenesis have yet to be elucidated. Therefore, conducting further research into the molecular drivers of *NPM1*-mutated AML pathogenesis can lead to new targeted strategies. As recommended, mutational screening of *FLT3*-ITD, *DNMT3A*, *IDH1/2,* and *TET2* could help guide clinicians in selecting the appropriate targeted treatment strategies for patients.

Currently, there are many targeted therapies, but due to the heterogeneity of *NPM1* mutants, treatment options will differ. Although the standard of care currently for AML is the “7 + 3” regimen, consideration is needed for *NPM1*-mutated AML co-mutated with; *FLT3*-ITD, for which FLT3 inhibitors are used or SYK inhibitors may also be used as in vivo studies have inferred SYK involvement in FLT3 activation. Other current treatment strategies include venetoclax and hypomethylating agents, Actinomycin D, and GO. Novel treatment strategies worth investigating is the use of daunorubicin, a popular anthracycline, in tandem with ATO and ATRA, menin inhibitors (due to the commonly seen overexpression of HOX cluster gene and XPO1 inhibitors linked to prolonged survival in mice studies. Lastly, immunotherapeutic approaches such as HLA-dependent T cell, CAR-T, and anti-PD-1 immunotherapy, should also be explored.

In addition, due to the wide array of suggested treatment strategies potential unwarranted side effects can occur. Therefore, further research on MRD monitoring strategies such as single-cell NGS and CyTOF on top of the typical qPCR and ddPCR may benefit in overall survival of patients and treatment management.

## Data Availability

Not applicable.
